# The effect of the formyl group position upon asymmetric isomeric diarylethenes bearing a naphthalene moiety

**DOI:** 10.3762/bjoc.8.114

**Published:** 2012-07-05

**Authors:** Renjie Wang, Shouzhi Pu, Gang Liu, Shiqiang Cui

**Affiliations:** 1 Jiangxi Key Laboratory of Organic Chemistry, Jiangxi Science & Technology Normal University, Nanchang 330013, PR China

**Keywords:** chemical diversity, diarylethene, electrochemistry, formyl group, photochromism, substituent position effect

## Abstract

Three new isomeric asymmetric diarylethenes with a naphthyl moiety and a formyl group at the para, meta or ortho position of the terminal benzene ring were synthesized. Their photochromism, fluorescent-switch, and electrochemical properties were investigated. Among these diarylethenes, the one with a formyl group at the ortho position of benzene displayed the largest molar absorption coefficients and fluorescence quantum yield. The cyclization quantum yields of these compounds increased in the order of para < ortho < meta, whereas their cycloreversion quantum yields decreased in the order of meta > para > ortho. Additionally, all of these diarylethenes functioned as effective fluorescent switches in both solution and PMMA films. Cyclic voltammograms proved that the formyl group and its position could effectively modulate the electrochemical behaviors of these diarylethene derivatives.

## Introduction

In the past decade, photochromic materials have received much attention because of their applications in potential photoswitchable, molecular devices and optical memory storage systems [1]. Among these materials, the utilization of diarylethene derivatives in molecular electronics, optical memory, and variable-transmission filters has been well documented [1–3]. So far, a huge number of studies concerning the photochromic properties of dithienylethene derivatives have been reported [4–14].

Light is a convenient and powerful trigger to control the reactivity of biomolecules in organisms, exemplified by its use in fluorescent probes, fluorescence imaging, and molecular switches in logic circuits [15–22]. In general, in order to efficiently realize the artificial induction of photosensitivity in biomolecules, the photoactive molecules must possess the following properties: (1) low cytotoxicity, (2) high sensitivity, (3) easy chemical modification. In the past few decades, various types of photochromic molecules, such as fulgides, spiropyranes, azobenzenes, and diarylethenes, have been developed [2,23–31].

Among these compounds, diarylethene is one of the most promising photoswitchable units within the photochromic system, and the successful use of diarylethenes as a fluorescence modulation center to realize a photoswitchable probe for imaging living cells was reported. For example, Zou et al. reported an amphiphilic molecule with hydrophilic and hydrophobic chains on two ends of a rigid diarylethene core. This compound can form stable vesicle nanostructures in aqueous solution, and exhibits excellent switchable fluorescence between open and closed states in the living cells, with low cytotoxicity [32]. Piao et al. developed a multiresponsive fluorescent molecular switch containing terpyridine. This diarylethene can serve as a detector for metal-ion transmembrane transport [33]. Singer et al. explored a novel diarylethene with a 7-deazaadenosine, which led to new research in photochromic nucleosides and molecular recognition properties of nucleic acids with the light sensitivity of diarylethenes [34]. Recently, Wu et al. designed and synthesized a novel diarylethene-containing dithiazolethene, which exhibited a gated photochromic reactivity controlled by complexation/dissociation with BF_3_ [35]. All of the above research revealed that the explored novel diarylethenes have versatile applications and are still important and attractive.

**Scheme 1:**
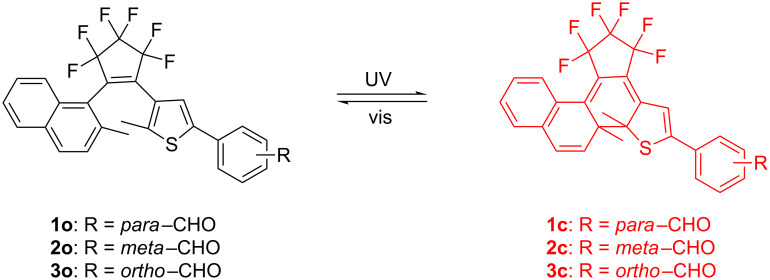
Photochromism of diarylethenes **1**–**3**.

Diarylethene derivatives, especially those containing a perfluorocyclopentene bridge are one of the most promising photochromic compounds due to their high fatigue resistance and thermal stability [2,36]. In general, the photochromic reactivity of diarylethenes mainly depends on heteroaryl groups and different electron-donor/acceptor substituents. The formyl group can be modified to form various chemical groups and it can also be connected with different fluorophores via a Schiff base structure. In a previous work, we developed a new class of diarylethenes with a naphthalene group and a thiophene group. The results revealed that these molecules have excellent photochromism with good fatigue resistance and thermal stability [37]. In this study, in order to further elucidate the substituent position effects on the photochromic features of naphthalene-containing diarylethenes, we synthesized three new isomeric diarylethenes with a formyl group at the para, meta, and ortho position on the terminal benzene ring (**1–3**). The photochromic scheme of **1–3** is shown in Scheme 1.

## Results and Discussion

**Scheme 2:**
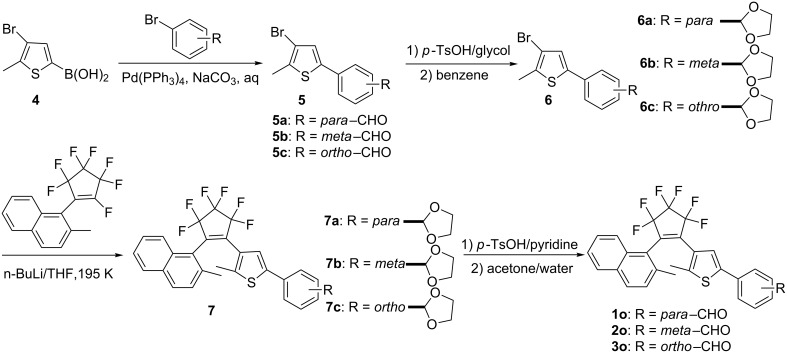
Synthetic route for diarylethenes **1**–**3**.

The synthesis route for diarylethenes **1o–3o** is shown in Scheme 2. First, the benzaldehydethiophene derivatives **5a–c** were prepared by Suzuki coupling of three bromobenzaldehyde derivatives with a thiopheneboronic acid **4** [38–42]. Second, 1,3-dioxolane-phenylthiophene derivatives **6a–c** were prepared by the reported method [39–41,43]. Then, 1,3-dioxolane-phenylthiophene derivatives **6a–c** were separately lithiated and coupled with (2-methylnaphth-1-yl)perfluorocyclopentene [37] to give the asymmetric diarylethene derivatives **7a–c** [44]. Finally, compounds **1o–3o** were prepared by hydrolyzing compounds **7a–c** in the presence of pyridine and *p*-toluenesulfonic acid in acetone/water. The structures of **1o–3o** were confirmed by elemental analysis, NMR, and IR (Supporting Information File 1).

### Photoisomerization of diarylethenes **1**–**3**

**Figure 1:**
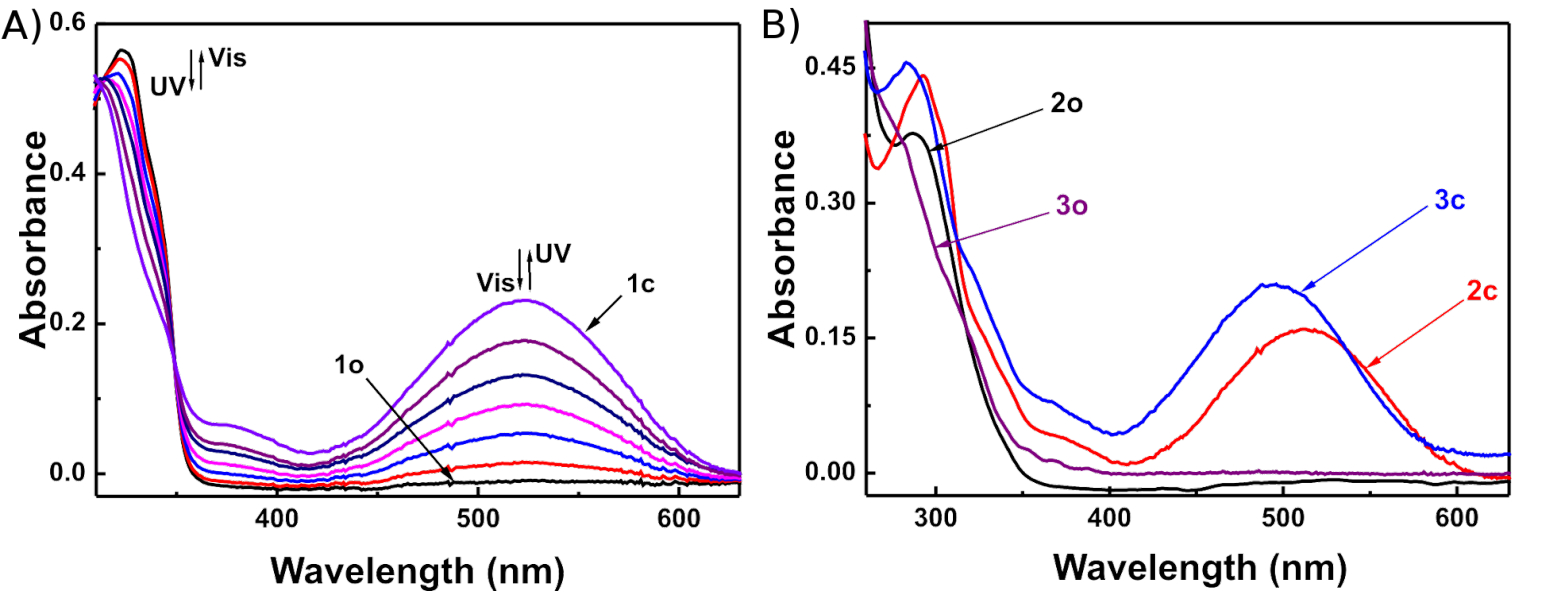
Absorption spectral changes of diarylethenes **1**–**3** by photoirradiation with UV–vis in hexane (2.0 × 10^−5^ mol/L) at room temperature: (A) **1**; (B) **2** and **3**.

**Figure 2:**
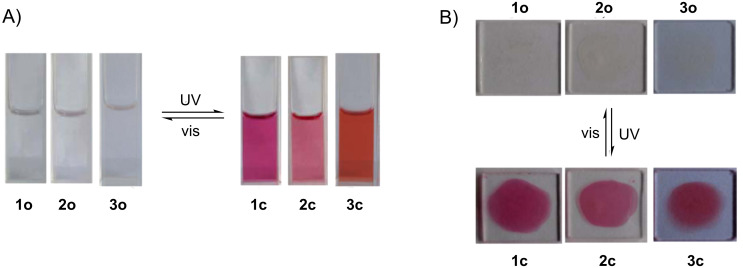
The color changes of diarylethene **1**–**3** by photoirradiation at room temperature: (A) in hexane; (B) in PMMA films.

Diarylethenes **1**−**3** showed good photochromic properties and could be toggled between their colorless open-ring isomers (**1o**−**3o**) and colored closed-ring isomers (**1c**−**3c**) by alternate irradiation with UV and visible light (λ > 500 nm). As shown in Figure 1A, diarylethene **1o** exhibited a sharp absorption peak at 323 nm (ε, 2.82 × 10^4^ L mol^−1^ cm^−1^) in hexane, which arose from the π→π* transition [45]. Upon irradiation with 297 nm light, the colorless solution of **1o** gradually turned red, and a new absorption band was observed in the visible region centered at 524 nm (ε, 1.40 × 10^4^ L mol^−1^ cm^−1^) due to the formation of the closed-ring isomer **1c**. Alternatively, the red-colored solution could be bleached to become colorless by re-production of the open-ring isomer **1o** upon irradiation with visible light (λ > 500 nm). In the photostationary state, a clear isosbestic point of diarylethene **1** was observed at 349 nm, which supported the reversible two-component photochromic reaction scheme [46]. Similarly **1o**, compounds **2o** and **3o** also showed good photochromism in hexane (Figure 1B). The colorless solutions of **2o** and **3o** turned pink and magenta due to the formation of the closed-ring isomers **2c** and **3c**, when irradiated with 297 nm light. Their absorption maxima appeared at 512 and 496 nm respectively. The colored solutions of **2c** and **3c** can also be decolorized upon irradiation with visible light (λ > 500 nm), and their isosbestic points were observed at 277 and 268 nm, respectively. The color changes of diarylethenes **1**–**3** by alternating irradiation with UV and visible light (λ > 500 nm) in hexane are shown in Figure 2A.

**Figure 3:**
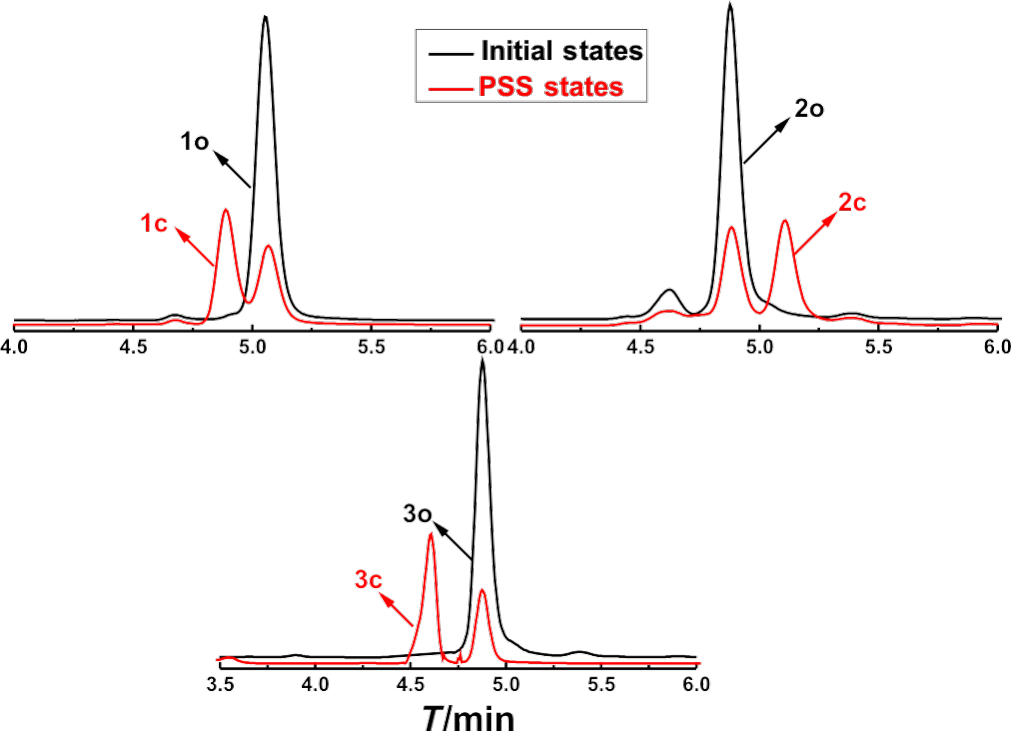
The photoconversion ratios of diarylethenes **1**–**3** in the photostationary state as analyzed by HPLC.

In PMMA amorphous films, diarylethenes **1**–**3** also showed similar photochromic activity to that in hexane. The absorption maxima of closed-ring isomers of diarylethenes **1c**–**3c** in PMMA films were at longer wavelengths. The values of the absorption maxima of the ring-closed isomers are 14 nm for **1c**, 7 nm for **2c**, and 21 nm for **3c**. The redshift phenomena may be ascribed to a polar effect of the polymer matrix and the stabilization of the molecular arrangement in the solid medium [47–48]. The color changes of diarylethenes **1**–**3** upon alternating irradiation with UV and visible light in PMMA films are shown in Figure 2B. The photoconversion ratios from open-ring to closed-ring isomers of **1**–**3** were analyzed by HPLC in the photostationary state (Figure 3). It was calculated that their photoconversion ratios in the photostationary state were 82% for **1**, 79% for **2**, and 81% for **3**.

The photochromic features of compounds **1**–**3** are summarized in Table 1. The results indicate that the position of the formyl group at the terminal benzene significantly affects the photochromic properties of these diarylethenes, such as the absorption maxima, molar absorption coefficients, and quantum yields of cyclization and cycloreversion. For the isomeric diarylethenes **1**–**3**, the absorption maxima of both the open-ring and closed-ring isomers exhibited a remarkable hypochromatic shift when the formyl group was moved from the para to the meta, then to the ortho position in both hexane and PMMA films, whereas the molar absorption coefficients of diarylethenes **1**–**3** increased in order of meta < para < ortho substitution by the formyl group in hexane. The results were in agreement with those of the reported diarylethenes containing an electron-withdrawing cyano group [49], but were different from those with an electron-donating methoxy group [14,50–51]. The cycloreversion quantum yields of diarylethenes **1**–**3** increased in the order of ortho (Φ_c-o_ = 0.11) < para (Φ_c-o_ = 0.12) < meta substitution (Φ_c-o_ = 0.15) by the formyl group. However, the cyclization quantum yield of the para-substituted derivative **1** was the largest (Φ_c-o_ = 0.35), while that of the meta-substituted derivative **2** was the lowest (Φ_c-o_ = 0.21). Compared to the electron-donating methoxy group or electron-withdrawing cyano group [49–50], the position of the electron-withdrawing formyl group can effectively modulate the absorption maxima of diarylethenes, which may be a novel strategy for exploring photochromic diarylethenes at shorter wavelengths.

**Table 1:** Absorption spectral properties of diarylethenes **1**–**3** in hexane (2.0 × 10^−5^ mol L^−1^) and in PMMA films (10%, w/w) at room temperature.

compound	λ_o,max_/nm^a^ (ε/L mol^−1^ cm^−1^)		λ_c,max_/nm^b^ (ε/L mol^−1^ cm^−1^)		Φ^c^		conversion at PSS in hexane

	hexane	PMMA film	hexane	PMMA film	Φ_o-c_	Φ_c-o_	

**1**	323 (2.82 × 10^4^)	326	524 (1.40 × 10^4^)	538	0.35	0.12	82
**2**	256 (2.75 × 10^4^)	288	512 (1.01 × 10^4^)	519	0.21	0.15	79
**3**	245 (3.48 × 10^4^)	248	496 (1.57 × 10^4^)	517	0.25	0.11	81

^a^Absorption maxima of ring-open isomers. ^b^Absorption maxima of ring-closed isomers. ^c^Quantum yields of ring-open (Φ_o-c_) and ring-closed isomers (Φ_c-o_), respectively.

**Figure 4:**
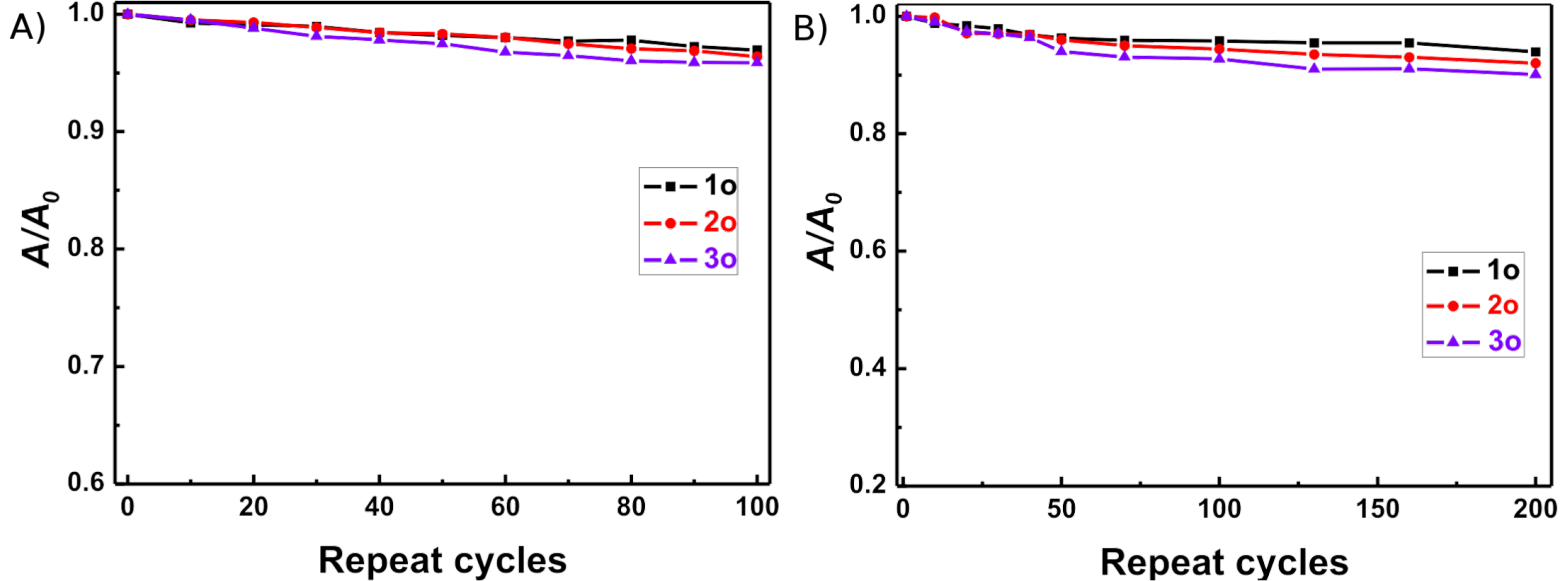
Fatigue resistance of diarylethenes **1**–**3** in hexane in air atmosphere at room temperature: (A) in hexane; (B) in PMMA films. Initial absorbance of the sample was fixed to 1.0.

The thermal stabilities of the open-ring and closed-ring isomers of **1**–**3** were tested by storing the compounds at both room temperature in hexane and at 351 K in ethanol. The hexane solutions were kept at room temperature in the dark, and exposed to air for more than two months. No changes in the UV–vis spectra were observed for **1**–**3**. At 351 K, diarylethenes **1**–**3** also showed excellent thermal stability for more than 12 h in ethanol. Fatigue resistance is a critical factor for practical applications in optical devices, and the fatigue resistances of diarylethenes **1**–**3** were examined in both hexane and PMMA films by alternate irradiation with UV and visible light at room temperature [2,52]. As shown in Figure 4, the coloration and decoloration cycle of **1**–**3** can be repeated more than 100 times in hexane with less than 5% degradation of **1c**–**3c**. In PMMA films, **1**–**3** also exhibited excellent photochromic properties after 200 cycles with only ca. 6–10% degradation of **1c**–**3c**. The results showed that all three isomeric diarylethenes **1**–**3** had good fatigue resistance in both hexane and PMMA films.

### Fluorescence of diarylethenes **1**–**3**

**Figure 5:**
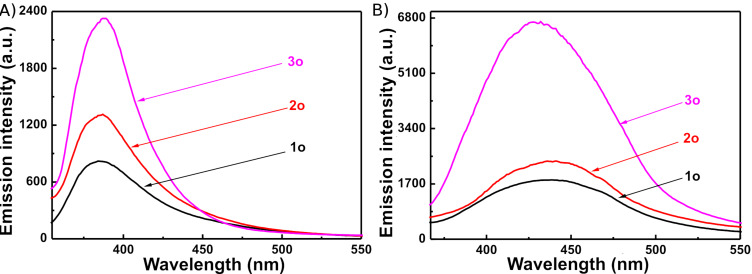
Fluorescence emission spectra of diarylethenes **1–3** at room temperature: (A) in hexane solution (2.0 × 10^−5^ mol L^−1^); (B) in PMMA films (10%, w/w).

Fluorescence can be used not only in molecular-scale optoelectronics but also in digital photoswitching [22,53–54]. Like most of reported diarylethenes, diarylethenes **1**–**3** exhibited notable fluorescence in both hexane and PMMA films. Their fluorescence spectra were measured at room temperature with a Hitachi F-4500 spectrophotometer (Figure 5). In hexane, the emission peaks of **1o**–**3o** were observed at 384, 387, and 389 nm, when excited at 307, 315, and 300 nm, whereas those in PMMA films were observed at 438, 441, and 427 nm, when excited at 334, 300, and 300 nm respectively. In comparison with those of **1o**–**3o** in hexane, the fluorescence emission peaks of **1o**–**3o** in PMMA films consistently exhibited a remarkable bathochromic shift. The emission intensity of the ortho-substituted derivative **3o** was the strongest, while that of the para-substituted derivative **1o** was the weakest in both hexane and PMMA films. Compared to the unsubstituted parent diarylethene, 1-(2-methylnaphth-1-yl)-2-[2-methyl-5-phenylthien-3-yl]perfluorocyclopentene (Φ_f_ = 0.011) [37], the fluorescence emission intensities of diarylethenes **1o** and **2o** were decreased, but that of **3o** was evidently increased. When anthracene was used as the reference, the fluorescence quantum yields of **1o**–**3o** were determined to be 0.012, 0.018, and 0.059, respectively, indicating that the formyl group on the terminal benzene ring could notably enhance the fluorescence quantum yield and remarkably influence the fluorescence emission intensity of diarylethenes with a naphthalene moiety.

**Figure 6:**
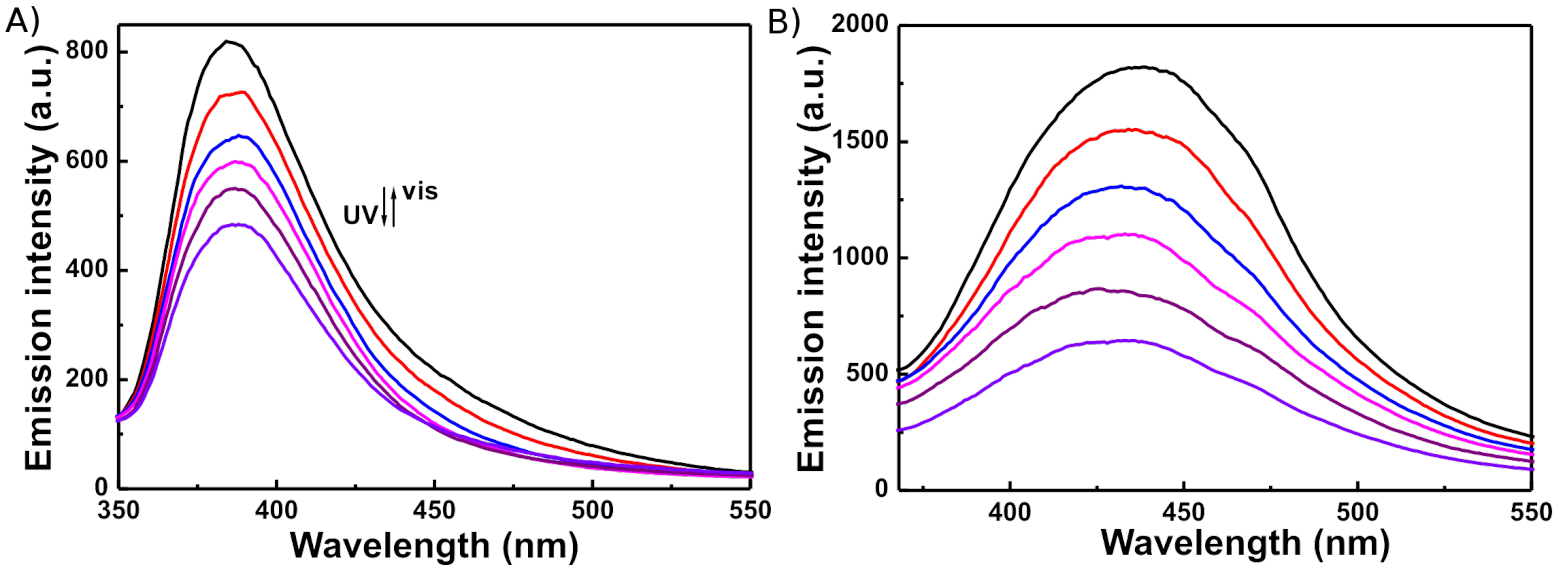
Emission intensity changes of diarylethene **1** upon irradiation with UV light at room temperature: (A) in hexane (excited at 307 nm), (B) in a PMMA film (excited at 324 nm).

Diarylethenes **1**–**3** exhibited an evident fluorescence switching capability upon changing from the open-ring to the closed-ring isomers by photoirradiation in both hexane and PMMA films. When irradiated by UV light, the photocyclization reaction yielded the nonfluorescent closed-ring isomers **1c**–**3c**, resulting in a decrease in emission intensity as compared to the open-ring isomers **1o**–**3o**. The back irradiation by visible light of appropriate wavelength (λ > 500 nm) regenerated the open-ring isomers **1o**–**3o**, and recovered their original emission intensity. As shown in Figure 6, upon irradiation with UV light, the emission intensity of **1** decreased, and was quenched to ca. 59% in hexane and 35% in a PMMA film, when it arrived at the photostationary state. The fluorescent modulation efficiency in the photostationary state was 41% in hexane and 65% in a PMMA film. Similarly, in the photostationary state, the fluorescence modulation efficiencies of diarylethenes **2** and **3** in hexane were 73 and 78%, and those in PMMA films were 38 and 42%, respectively. The residual fluorescence for **1**–**3** in the photostationary state may be attributed to an incomplete cyclization reaction and the existence of parallel conformations [55–56]. Among the three isomeric derivatives, the fluorescent modulation efficiency of diarylethene **2** was the largest and that of **1** was the smallest in PMMA films, suggesting that the diarylethene **2** is the best candidate for the fluorescence photoswitching material.

### Electrochemical properties of diarylethenes **1**–**3**

**Figure 7:**
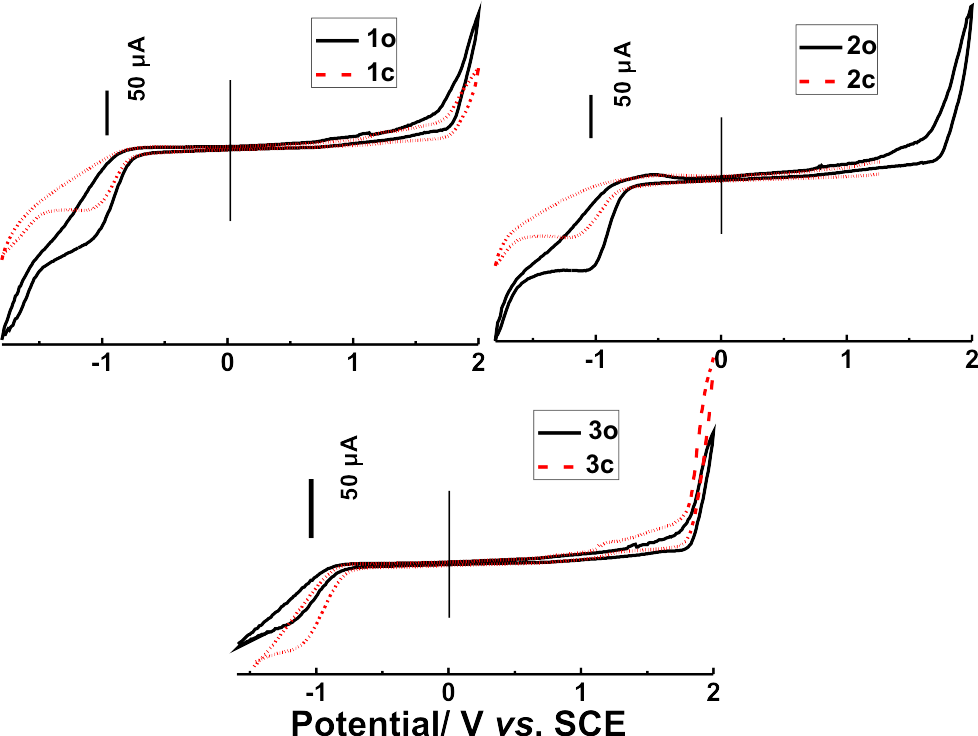
Cyclic voltammetry of diarylethenes **1–3** in acetonitrile with a scanning rate of 50 mV/s.

The electrochemical behaviors of diarylethene derivatives have attracted much attention because of their potential applications in molecular-scale electronic switches [57–62]. The electrochemical properties of **1**–**3** were evaluated by cyclic voltammetry (CV) under the same experimental conditions reported previously [13]. The CV curves of diarylethenes **1**–**3** are shown in Figure 7. The onset potentials (*E*_onset_) of oxidation and reduction for **1o** were initiated at +1.79 and −0.89 V, and those of **1c** at +1.76 and −0.91 V, respectively. According to the reported method [63–64], the ionization potential and electron affinities of **1o** were calculated to be −6.59 and −4.09 eV, and those of **1c** were −6.56 and −3.89 eV. Based on the highest occupied molecular orbital (HOMO) and lowest unoccupied molecular orbital (LUMO) energy level, the band gap *E*_g_ (*E*_g_ = LUMO–HOMO) of **1o** and **1c** can be determined to be +2.50 and +2.67 eV. Similarly, the oxidation potential of **2o** and **3o** is initiated at +1.76 and +1.83 V, and that of **2c** and **3c** is initiated at +1.74 and +1.79 V. The results indicate that the oxidation process for the open-ring isomers **1o–3o** occurs at higher potentials than in the corresponding closed-ring isomers **1c–3c**. This was because the longer conjugation length of the closed-ring isomers generally leads to a less positive potential [62,65]. The cyclization reaction allows the π-conjugation to extend across the perfluorocyclopentene ring causing a lower oxidation onset. As shown in Table 2, for the band gap of diarylethenes **1** and **2**, the values of *E*_g_ of the open-ring isomers were lower than those of the closed-ring isomers, with the exception of **3**. Among these compounds, the *E*_g_ of **1o** was the smallest, which implies that the charge transfer of **1o** was faster compared to that in others [66]. All these data suggest that the position of the formyl group at the terminal benzene ring has a remarkable effect on the electrochemical behaviors of these diarylethenes, but further work is required to quantify these effects.

**Table 2:** Electrochemical properties of diarylethenes **1**–**3**.

compound	oxidation		reduction		band gap

	*E*_onset_ (V)	IP (eV)	*E*_onset_ (V)	EA (eV)	*E*_g_

**1o**	+1.79	−6.59	−0.89	−4.09	2.50
**1c**	+1.76	−6.56	−0.91	−3.89	2.67
**2o**	+1.76	−6.56	−0.83	−3.97	2.59
**2c**	+1.74	−6.54	−1.01	−3.79	2.75
**3o**	+1.83	−6.63	−0.87	−3.93	2.70
**3c**	+1.79	−6.59	−0.80	−4.00	2.59

## Conclusion

Three new asymmetric isomeric diarylethenes containing a formyl group at either the para, meta, or ortho position of the terminal benzene ring were synthesized for the investigation of the effect of substituent position on their optical and electrochemical properties. The results revealed that the formyl group and its position had significant effects on the properties of these isomeric diarylethenes. The electron-withdrawing formyl group endowed these diarylethenes with some new properties, which were different from those of diarylethenes with a methoxy group or halide at the terminal benzene.

## Supporting Information

File 1 Experimental procedures and spectral data.
