# Supplementary material for: The effect of the formyl group position upon asymmetric isomeric diarylethenes bearing a naphthalene moiety
Source: Beilstein J Org Chem. 2012 Jul 5;8:1018–26. doi: 10.3762/bjoc.8.114 (PMC3458719; doi:10.3762/bjoc.8.114)
Supplement: File 1 — Experimental procedures and spectral data. [file Beilstein_J_Org_Chem-08-1018-s001.pdf]

Supporting Information

for

The effect of the formyl group position upon asymmetric isomeric diarylethenes bearing a naphthalene moiety

Renjie Wang, Shouzhi Pu*[,§], Gang Liu and Shiqiang Cui

Address: Jiangxi Key Laboratory of Organic Chemistry, Jiangxi Science & Technology Normal University, Nanchang 330013, PR China

E-mail address: Shouzhi Pu - pushouzhi@tsinghua.org.cn

* Corresponding author:

[§]Tel.: +86-791-83831996; Fax: +86-791-83831996.

Experimental procedures and spectral data

Table of Contents

1. General experimental details.. S2

2. Synthesis and characterizations S3

1. General experimental details

All solvents used were spectroscopic grade and were purified by distillation before use. NMR spectra were recorded on a Bruker AV400 (400 MHz) spectrometer with $CDCl_3$ as the solvent and tetramethylsilane as an internal standard. Infrared spectra (IR) were recorded on a Bruker Vertex-70 spectrometer. Melting points was taken on a WRS-1B melting point apparatus. Absorption spectra were measured using an Agilent 8453 UV–vis spectrophotometer. Photoirradiation was carried out using an SHG-200 UV lamp, a CX-21 ultraviolet fluorescence analysis cabinet and a BMH-250 visible lamp. The required wavelength was isolated by the use of the appropriate filters. Fluorescence spectra were measured using a Hitachi F-4500 spectrophotometer. Electrochemical examinations were performed in a one-compartment cell by using a Model 263 potentiostat–galvanostat (EG&G Princeton Applied Research) under computer control at room temperature. Platinum wire (diameter 0.5 mm) and steel electrodes served as working and counter electrodes, respectively. Platinum wire (diameter 0.5 mm) in the supporting electrolyte solution served as a quasi-reference electrode, which was calibrated using an internal ferrocene (Fc/Fc^+) standard with a formal potential of $E_{1/2}$ = +0.35 V versus platinum wire in the same electrolyte. The typical electrolyte was acetonitrile (5 mL) containing 0.1 mol/L tetrabutylammonium tetrafluoroborate ($(TBA)BF_4$) and 1.0×10^{-3} mol/L diarylethene sample. All solutions were deaerated by bubbling with a dry argon stream and maintained at a slight argon overpressure during electrochemical experiments.

2. Synthesis and characterizations

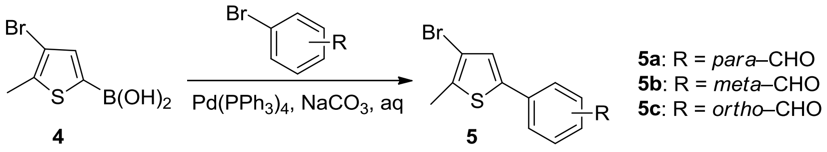

3-Bromo-2-methyl-5-(4-formylphenyl)thiophene (5a)

Compound **5a** was prepared by reacting (3-bromo-2-methylthien-5-yl)boronic acid (**4**) [1] (2.5 g, 11.3 mmol) with 4-bromobenzaldehyde (2.1 g, 11.3 mmol) in the presence of $Pd(PPh_3)_4$ (0.15 g, 0.01 mmol) and Na_2CO_3 (2 mol/L, 50 mL) in tetrahydrofuran (THF) (120 mL). After being heated under reflux for 16 h, the organic layer was dried over $MgSO_4$, filtered and evaporated. The crude product was purified by column chromatography on SiO_2 using (petroleum ether/ethyl acetate 6:1) as eluent to give **5a** [2] (2.2 g, 69%) as a white solid. Mp 387–388 K; ^{1}H NMR (400 MHz, $CDCl_3$, TMS) δ 2.44 (s, 3H, $–CH_3$), 7.24 (s, 1H, thiophene–H), 7.65 (d, J = 8.0 Hz, 2H, benzene–H), 7.87 (d, J = 8.0 Hz, 2H, benzene–H), 9.98 (s, 1H, formyl–H).

3-Bromo-2-methyl-5-(3-formylphenyl)thiophene (5b)

Compound **5b** [3] was prepared by an analogous method to that used for **5a** and was obtained as a light-yellow solid in 72% yield. Mp 383–384 K; ^{1}H NMR (400 MHz, $CDCl_3$, TMS) δ 2.44 (s, 3H, $–CH_3$), 7.20 (s, 1H, thiophene–H), 7.54 (t, J = 8.0 Hz, 1H, benzene–H), 7.77 (t, J = 8.0 Hz, 2H, benzene–H), 8.00 (s, J = 8.0 Hz, 1H, benzene–H), 10.04 (s, 1H, formyl–H).

3-Bromo-2-methyl-5-(2-formylphenyl)thiophene (5c)

Compound **5c** [4] was prepared by an analogous method to that used for **5a** and obtained as a yellow solid in 76% yield. Mp 332–333 K; ^{1}H NMR (400 MHz, $CDCl_3$, TMS) δ 2.46 (s, 3H, $–CH_3$), 6.89 (s, 1H, thiophene–H), 7.46–7.51 (m, 2H, benzene–H), 7.61 (t, J = 8.0 Hz,

1H, benzene–H), 7.99 (d, *J* = 8.0 Hz, 1H, benzene–H), 10.21 (s, 1H, formyl–H).

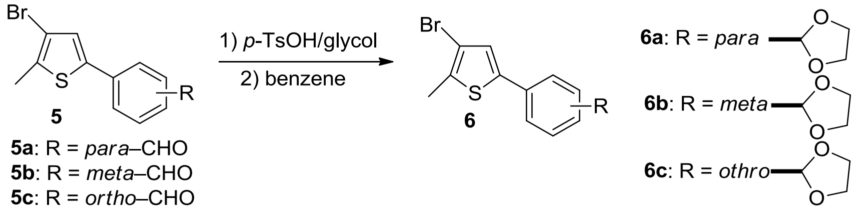

3-Bromo-2-methyl-5-(4-(1,3-dioxolan-2-yl)phenyl)thiophene (6a)

Compound **5a** (1.2 g, 4.27 mmol), glycol (1.5 mL, 27.1 mmol) and *p*-toluenesulfonic acid (0.002 g) were dissolved in benzene (120 mL). Under the Dean–Stark condition, the reaction mixture was heated under reflux overnight, and then washed with aqueous $NaHCO_3$ (5% (w/v), 2 × 50 mL). The combined benzene layers were dried, filtered and evaporated in vacuum to yield **6a** [2] as a yellow crystal (1.3 g, 93%). Mp 411–412 K; ^{1}H NMR (400 MHz, $CDCl_3$, TMS) δ 2.41 (s, 3H, $–CH_3$), 3.94–4.15 (m, 4H, 1,3-dioxolane–H), 5.81 (s, 1H, 1,3-dioxolane–H), 7.12 (s, 1H, thiophene–H), 7.46–7.53 (m, 4H, benzene–H).

3-Bromo-2-methyl-5-(3-(1,3-dioxolan-2-yl)phenyl)thiophene (6b)

Compound **6b** was prepared by an analogous method similar to that used for **6a** and was obtained as a pale yellow oil in 93% yield. ^{1}H NMR (400 MHz, $CDCl_3$, TMS) δ 2.41 (s, 3H, $–CH_3$), 3.94–4.16 (m, 4H, 1,3-dioxolane–H), 5.78 (s, 1H, 1,3-dioxolane–H), 6.93 (s, 1H, thiophene–H), 7.28–7.40 (m, 2H, benzene–H), 7.63 (d, *J* = 8.0 Hz, 1H, benzene–H), 7.73 (s, *J* = 8.0 Hz, 1H, benzene–H).

3-Bromo-2-methyl-5-(2-(1,3-dioxolan-2-yl)phenyl)thiophene (6c)

Compound **6c** [4] was prepared by an analogous method similar to that used for **6a**

andwas obtained as a yellow solid in 94% yield. Mp 349–350 K; ^{1}H NMR (400 MHz, $CDCl_3$, TMS) δ 2.43 (s, 3H, $–CH_3$), 4.02–4.21 (m, 4H, 1,3-dioxolane–H), 5.87 (s, 1H, 1,3-dioxolane–H), 7.06 (s, 1H, thiophene–H), 7.38–7.43 (m, 3H, benzene–H), 7.73 (d, *J* = 8.0 Hz, 1H, benzene–H).

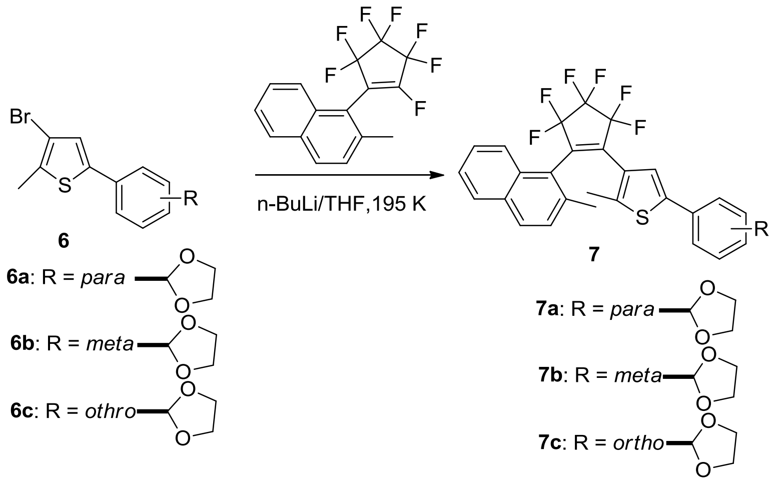

1-(2-Methylnaphth-1-yl)-2-[2-methyl-5-(4-(1,3-dioxolan-2-yl)phenyl)thien-3-yl]per-fluorocyclopentene (7a)

To stirred anhydrous THF containing **6a** (0.78 g, 2.4 mmol) was added dropwise a 2.4 mol/L *n*-BuLi/hexane solution (1.3 mL, 3.12 mmol) at 195 K under an argon atmosphere. After the mixture has been stirred for 30 min, compound (2-methylnaphth-1-yl)perfluorocyclopentene (0.8 g, 2.4 mmol) in anhydrous THF was added. The reaction was further stirred at 195 K for 2 h, and the reaction mixture was allowed to slowly warm to the room temperature. The reaction was quenched with distilled water. The product was extracted with diethyl ether, dried with $MgSO_4$, and concentrated under reduced pressure. The crude product was purified by column chromatography using (petroleum ether/ethyl acetate 6:1) as the eluent to afford 0.45 g of compound **7a** [5]

as a light-yellow solid in 35% yield. ^{1}H NMR (400 MHz, $CDCl_3$, ppm) δ 2.23 (s, 3H, $-CH_3$), 2.34 (s, 3H, $-CH_3$), 4.01–4.12 (m, 4H, 1,3-dioxolane–H), 5.79 (s, 1H, 1,3-dioxolane–H), 6.83 (s, 1H, thiophene−H), 7.17 (d, *J* = 8.0 Hz, 1H, benzene−H), 7.30−7.34 (m, 3H, benzene−H), 7.43−7.62 (m, 3H, naphthalene−H), 7.69 (d, *J* = 8.0 Hz, 1H, naphthalene−H), 7.78 (t, *J* = 8.0 Hz, 2H, naphthalene−H).

1-(2-Methylnaphth-1-yl)-2-[2-methyl-5-(3-(1,3-dioxolan-2-yl)phenyl)thien-3-yl]per-fluorocyclopentene (7b)

Compound **7b** was prepared by an analogous method to that used for **7a** and was obtained as a yellow oil in 20% yield. ^{1}H NMR (400 MHz, $CDCl_3$, ppm) δ 2.16 (s, 3H, $-CH_3$), 2.34 (s, 3H, $-CH_3$), 4.01–4.15 (m, 4H, 1,3-dioxolane–H), 5.57 (s, 1H, 1,3-dioxolane–H), 6.88 (s, 1H, thiophene−H), 7.19 (d, *J* = 8.0 Hz, 1H, benzene−H), 7.30−7.39 (m, 3H, benzene−H), 7.42−7.54 (m, 3H, naphthalene−H), 7.70 (d, *J* = 8.0 Hz, 1H, naphthalene−H), 7.81 (t, *J* = 8.0 Hz, 2H, naphthalene−H).

1-(2-Methylnaphth-1-yl)-2-[2-methyl-5-(3-(1,3-dioxolan-2-yl)phenyl)thien-3-yl]per-fluorocyclopentene (7c)

Compound **7c** was prepared by an analogous method to that used for **7a** and was obtained as a yellow oil in 28% yield. ^{1}H NMR (400 MHz, $CDCl_3$, ppm) δ 2.12 (s, 3H, $-CH_3$), 2.36 (s, 3H, $-CH_3$), 3.84–3.92 (m, 2H, 1,3-dioxolane–H), 4.06–4.13 (m, 2H, 1,3-dioxolane–H), 5.51 (s, 1H, 1,3-dioxolane–H), 6.95 (s, 1H, thiophene−H), 7.16 (d, *J* = 8.0 Hz, 1H, benzene−H), 7.27−7.35 (m, 3H, benzene−H), 7.43−7.51 (m, 2H, naphthalene−H), 7.63−7.70 (m, 2H, naphthalene−H), 7.81 (d, *J* = 8.0 Hz, 2H, naphthalene−H).

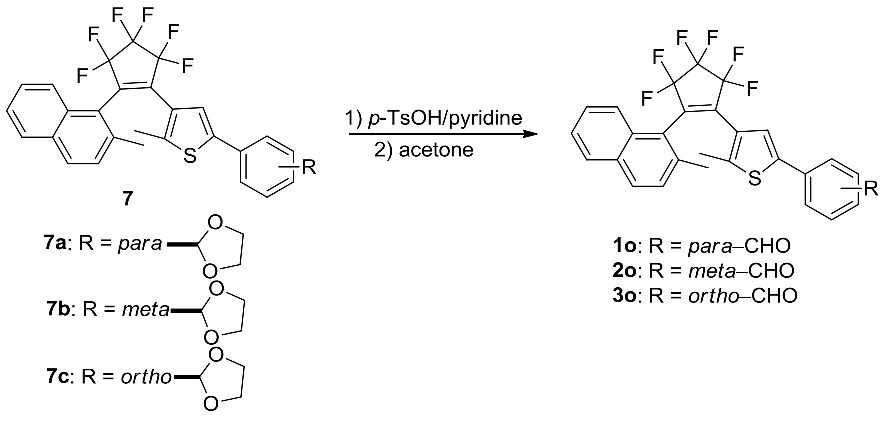

1-(2-Methylnaphth-1-yl)-2-[2-methyl-5-(4-formylphenyl)thien-3-yl]perfluorocyclo-pentene (1o)

Compound **7a** (0.45 g, 8.0 mmol), pyridine (0.635 g, 8.0 mmol) and *p*-toluenesulfonic acid (8.0 mmol, 1.52 g) were dissolved in a mixture of acetone (50 mL) and water (10 mL). The reaction mixture was heated under reflux overnight at 333 K, and then washed with $NaHCO_3$ (10% (w/v), 2 × 20 mL) aqueous and water. The resultant mixture was then extracted with ether, and the organic extract was washed with brine and dried ($MgSO_4$). The solvent was removed evaporated in vacuum, and the residue was purified by column chromatography on silica gel to yield **1o** as a yellow powder solid (0.45 g, 98%). Mp 457–458 K; Anal. calcd for $C_{28}H_{18}F_6OS$: C, 65.11; H, 3.51; found C, 65.07; H, 3.47; ^{1}H NMR (400 MHz, $CDCl_3$, ppm) δ 2.23 (s, 3H, $-CH_3$), 2.36 (s, 3H, $-CH_3$), 6.98 (s, 1H, thiophene−H), 7.32 (t, *J* = 8.0 Hz, 3H, benzene−H), 7.47−7.57 (m, 2H, naphthalene−H), 7.69−7.70 (m, 1H, benzene−H), 7.75 (d, *J* = 8.0 Hz, 2H, naphthalene−H), 7.84 (t, *J* = 8.0 Hz, 2H, naphthalene−H); 9.93 (s, 1H, formyl−H); ^{13}C NMR (100 MHz, $CDCl_3$, TMS) δ 14.95, 20.42, 114.21, 123.18, 124.22, 124.81, 125.43, 125.57, 125.69, 126.34, 127.06, 127.60, 128.35, 128.52, 130.11, 130.33, 131.83, 135.17, 135.45, 138.82, 139.48, 142.96, 191.16; IR (KBr, *v*, cm^{-1}): 516, 744, 771, 811, 824, 871, 893, 984, 1048, 1107, 1132, 1172,

1191, 1274, 1338, 1437, 1601, 1695, 2745, 3282; HRMS *m*/*z* (M^+) = 516.1003 (calcd 516.0983).

1-(2-Methylnaphth-1-yl)-2-[2-methyl-5-(3-formylphenyl)thien-3-yl]perfluorocyclo-pentene (2o)

Compound **2o** was prepared by an analogous method to that used for **1o** and was obtained as a yellow solid in 90% yield. Mp 325–326 K; Anal. calcd for $C_{28}H_{18}F_6OS$: C, 65.11; H, 3.51; found C, 65.14; H, 3.61; ^{1}H NMR (400 MHz, $CDCl_3$, ppm) δ 2.21 (s, 3H, $-CH_3$), 2.35 (s, 3H, $-CH_3$), 6.93 (s, 1H, thiophene−H), 7.33 (d, *J* = 8.0 Hz, 1H, benzene−H), 7.43 (d, *J* = 8.0 Hz, 2H, benzene−H), 7.49−7.55 (m, 2H, naphthalene−H), 7.62 (s, 1H, benzene−H), 7.69 (d, *J* = 8.0 Hz, 2H, naphthalene−H), 7.82 (t, *J* = 8.0 Hz, 2H, naphthalene−H), 9.93 (s, 1H, formyl−H); ^{13}C NMR (100 MHz, $CDCl_3$, TMS) δ 14.87, 20.41, 123.28, 124.23, 124.44, 124.88, 125.13, 125.68, 126.16, 127.04, 128.34, 128.54, 128.60, 129.55, 130.09, 130.85, 131.72, 134.25, 135.48, 136.68, 139.46, 141.15, 141.90, 191.66; IR (KBr, *v*, cm^{-1}): 512, 559, 647, 680, 742, 771, 811, 871, 895, 980, 1048, 1131, 1192, 1273, 1336, 1494, 1706, 2734, 3281; HRMS *m*/*z* (M^+) = 516.1005 (calcd 516.0983).

1-(2-Methylnaphth-1-yl)-2-[2-methyl-5-(2-formylphenyl)thien-3-yl]perfluorocyclo-pentene (3o)

Compound **3o** was prepared by an analogous method to that used for **1o** and was obtained as a yellow solid in 60% yield. Mp 371–372 K; Anal. calcd for $C_{28}H_{18}F_6OS$: Calcd C, 65.11; H, 3.51; found C, 65.05; H, 3.59; ^{1}H NMR (400 MHz, $CDCl_3$, ppm) δ 2.33 (s, 3H, $-CH_3$), 2.38 (s, 3H, $-CH_3$), 6.53 (s, 1H, thiophene−H), 7.27 (s, 1H, benzene−H), 7.39 (t, *J* = 8.0 Hz, 2H, benzene−H), 7.43 (d, *J* = 8.0 Hz, 2H, benzene−H), 7.47−7.53 (m, 3H,

naphthalene−H), 7.63 (d, J = 8.0 Hz, 1H, benzene−H), 7.84−7.87 (m, 3H, naphthalene−H), 9.34 (s, 1H, formyl−H); ^{13}C NMR (100 MHz, $CDCl_3$, TMS) δ 14.67, 20.44, 122.97, 124.43, 124.74, 125.71, 127.12, 127.94, 128.33, 128.39, 128.50, 129.76, 130.28, 130.80, 131.55, 131.83, 133.46, 133.74, 135.38, 135.83, 136.50, 143.06, 190.71; IR (KBr, ν, cm^{-1}): 514, 612, 624, 768, 814, 889, 979, 1130, 1192, 1265, 1334, 1400, 1638, 1686, 3414; HRMS *m*/*z* (M^+) = 516.0996 (calcd 516.0983).

References

[1] Li, Z. X.; Liao, L.-Y.; Sun, W.; Xu, C.-H.; Zhang, C.; Fang, C.-J.; Yan, C.-H. *J. Phys. Chem. C* **2008,** *112*, 5190–5196. doi: 10.1021/jp711613y

[2] Yamamoto, S.; Matsuda, K.; Irie, M. *Org. Lett.* **2003,** *5*, 1769–1772. doi:10.1021/ol034440h

[3] Yamamoto, S.; Matsuda, K.; Irie, M. *Angew. Chem., Int. Ed.* **2003,** *42*, 1636–1639. doi:10.1002/anie.200250417

[4] Yamamoto, S.; Matsuda, K.; Irie, M. *Chem.-Eur. J.* **2003,** *9*, 4878–4886. doi:10.1002/chem.200304947

[5] Wang, R. J.; Li, W. B.; Liu, W. J.; Liu,G. *Adv. Mater. Res.* **2012,** *393–395*, 381–384. doi:10.4028/www.scientific.net/AMR.393-395.381
